# Modeling Coastal Vulnerability through Space and Time

**DOI:** 10.1371/journal.pone.0163495

**Published:** 2016-10-12

**Authors:** Thomas Hopper, Marcia S. Meixler

**Affiliations:** Department of Ecology, Evolution, and Natural Resources, Rutgers University, New Brunswick, New Jersey, United States of America; University of Waikato, NEW ZEALAND

## Abstract

Coastal ecosystems experience a wide range of stressors including wave forces, storm surge, sea-level rise, and anthropogenic modification and are thus vulnerable to erosion. Urban coastal ecosystems are especially important due to the large populations these limited ecosystems serve. However, few studies have addressed the issue of urban coastal vulnerability at the landscape scale with spatial data that are finely resolved. The purpose of this study was to model and map coastal vulnerability and the role of natural habitats in reducing vulnerability in Jamaica Bay, New York, in terms of nine coastal vulnerability metrics (relief, wave exposure, geomorphology, natural habitats, exposure, exposure with no habitat, habitat role, erodible shoreline, and surge) under past (1609), current (2015), and future (2080) scenarios using InVEST 3.2.0. We analyzed vulnerability results both spatially and across all time periods, by stakeholder (ownership) and by distance to damage from Hurricane Sandy. We found significant differences in vulnerability metrics between past, current and future scenarios for all nine metrics except relief and wave exposure. The marsh islands in the center of the bay are currently vulnerable. In the future, these islands will likely be inundated, placing additional areas of the shoreline increasingly at risk. Significant differences in vulnerability exist between stakeholders; the Breezy Point Cooperative and Gateway National Recreation Area had the largest erodible shoreline segments. Significant correlations exist for all vulnerability (exposure/surge) and storm damage combinations except for exposure and distance to artificial debris. Coastal protective features, ranging from storm surge barriers and levees to natural features (e.g. wetlands), have been promoted to decrease future flood risk to communities in coastal areas around the world. Our methods of combining coastal vulnerability results with additional data and across multiple time periods have considerable potential to provide valuable predictions that resource managers can effectively use to identify areas for restoration and protection.

## Introduction

Coastal ecosystems experience a wide range of stressors including wave forces, storm surge, sea-level rise, and anthropogenic modification. Coastal ecosystems also absorb many of these stressors through wave attenuation and protection from erosion, thus providing valuable ecosystem services to people and property [1–5]. When stressors outweigh a coastal ecosystem’s ability to attenuate them, the coastal ecosystems may become vulnerable to erosion. When coastal ecosystems are lost to erosion their ecosystem services capacity is reduced, shifting environmental stressors to an often urban, developed inland. Urban coastal ecosystems are especially important due to the large populations these limited ecosystems serve. Within the United States, coastal areas have averaged about 54% of the nation’s population since 1960 [6]. Although coastal ecosystems are limited in scope in urban settings they are especially important due to the large populations they serve. For example, coastal wetlands in New York State are scarce compared to wetlands found in other states, but they have the greatest value at just over USD$ 5,100,000 per km^2^ per year due to large coastal populations, dense infrastructure, and high property value [5].

The recent natural history of Jamaica Bay, New York, situated on the southern shore of Long Island, is one of extensive anthropogenic modification through dredging of channels and filling of marshes [7]. According to the New York City Department of Environmental Protection (NYCDEP), while the surface area of the bay has been reduced from 101 km^2^ in the mid-nineteenth century to 53 km^2^ in 2007, the volume of the bay has increased 350% [8]. Most of the peripheral land in the bay consists of artificial fill [9]; the construction of Floyd Bennett Field alone involved over 396,000 m^3^ of fill [7]. Throughout most of the early twentieth century these activities were guided and supported by government agencies. Until the 1930’s, many of those agencies followed a plan for the development of Jamaica Bay into an ocean port and a commercial and industrial center. This plan called for the elimination of all marshes and meadows within the bay and the creation of two large and entirely bulkheaded islands with an extensive system of piers [7,10]. As of 2006, nearly 43% of the shoreline had been artificially modified through bulkheads, roads and other structures [11].

It is well documented that the marshes of Jamaica Bay are rapidly disappearing [9,12,13,14]. Saltmarshes covered an estimated 65.5 km^2^ in 1900 [15]. By 1970, only around 16.2 km^2^ of saltmarshes remained [9]. Causes for the wetland losses in Jamaica Bay are under debate but include sea-level rise [9], changes in tidal patterns due to dredging activities [14], high nutrient loading [13], and changes in the source/sink components of the sediment budget due to anthropogenic changes to bathymetry and upland development [12].

Traditional approaches for defending shorelines from erosion have relied heavily on armoring shorelines with engineered structures such as bulkheads [16]. These alterations often cause unplanned environmental impacts in the long term [17] such as significant decreases in biodiversity and species abundance [18]. One significant threat faced by marshes in Jamaica Bay is “coastal squeeze.” Coastal squeeze is a form of intertidal coastal habitat loss due to a shrinking intertidal zone. This occurs when the high water mark of an intertidal zone is fixed by a defense or other hard structure and the low water mark migrates landward in response to sea-level rise [19]. Since Jamaica Bay’s peripheral marshes are fringing, backed by extensive urbanization and infrastructure, they lack the opportunity for significant landward migration. Over time the marshes of Jamaica Bay may be squeezed due to sea-level rise, leaving previously sheltered structures to bear the brunt of storm surges and wave action. If Jamaica Bay’s marshes are lost, coastal defense infrastructure will need to be created and/or upgraded in order to avoid an increase in coastal vulnerability. While bulkheads and other coastal defense structures may compensate for lost wave attenuation services, they do not provide the array of other services coastal ecosystems do such as carbon sequestration, water treatment, wildlife habitat, or unique recreational opportunities.

The purpose of this study was to model and map coastal vulnerability and the role of natural habitats in reducing vulnerability in Jamaica Bay, New York in terms of nine coastal vulnerability metrics (relief, wave exposure, geomorphology, natural habitats, exposure, exposure with no habitat, habitat role, erodible shoreline, and surge) under past (1609), current (2015), and future (2080) scenarios. We analyzed vulnerability results spatially and across all time periods, by stakeholder (ownership) and by distance to damage from Hurricane Sandy. This study differs from others in that it evaluates coastal vulnerability and the reduction of vulnerability due to coastal natural habitats in a spatially explicit manner, at the landscape scale, with spatial data that are finely resolved, and in a location with high quality spatial data over multiple time periods. The importance of these characteristics in urban ecosystem studies has been noted by other researchers [1,3,20].

## Methods

**Fig 1:**
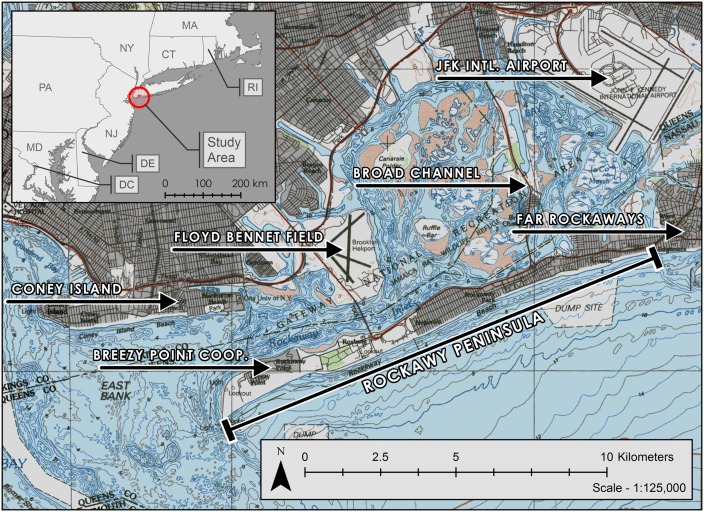
Study area Jamaica Bay, New York including relevant place names. Base map obtained from USGS: Long Island West, New York-New Jersey-Connecticut, Topographic-Bathymetric Map, 1984; USGS Sheet 40073-E1-TB-100. Inset map state boundaries obtained from U.S. Census Bureau, Second Edition TIGER/Line files—State Boundaries.

Our study area is Jamaica Bay, New York, (40°36'44"N 73°50'19"W) situated on the southern shore of Long Island, and characterized by extensive coastal ecosystems in the central bay juxtaposed with a largely urbanized shoreline containing fragmented and fringing coastal habitat. The bay is surrounded on three sides by the New York City Boroughs of Brooklyn and Queens, bounded by John F. Kennedy (JFK) International Airport and the Far Rockaways to the east, Floyd Bennett Field to the west, and the Rockaway Peninsula to the south (Fig 1). Broad Channel is the largest and only inhabited island in the bay, Coney Island lies just outside of the bay’s mouth, and the Breezy Point Cooperative lies near the tip of the Rockaway Peninsula (Fig 1). Approximately 56% of the bay’s shoreline is within the statutory boundary of the Jamaica Bay unit of the Gateway National Recreation Area (GNRA), administered by the National Park Service (NPS). The Congress of the United States created GNRA in 1972 (Public Law 92–592) and it is one of the most utilized national parks system units in the United States with over 10 million visits each year [21].

We used the Integrated Valuation of Ecosystem Services and Tradeoffs tool (InVEST) version 3.2.0 [22] coastal vulnerability model to assess coastal vulnerability in Jamaica Bay. InVEST is a site of free, open-source software models created by the Natural Capital Project [22]. This model calculates an exposure index by combining the ranks of six bio-geophysical variables for each shoreline segment: geomorphology, natural habitats, relief, wave exposure, wind exposure and surge. Ranks vary from very low exposure (rank = 1) to very high exposure (rank = 5) (Table 1).

**Table 1:** List of bio-geophysical variables and rankings.

Variable	Very low	Low	Moderate	High	Very high
	1	2	3	4	5
Geomorphology	Rocky, High cliff, Seawalls	Medium cliff, Bulkheads, Small seawalls	Low cliff, Alluvial plain, Rip-rap	Cobble Beach, Lagoon, Bluff	Barrier beach, Sandy beach, Mud flat
Natural Habitats	Coral reef, mangrove, High saltmarsh	High dune, Low marsh	Low dune, Scrubland	Seagrass, Kelp	No habitat
Relief	0 to 20 Percentile	21 to 40 Percentile	41 to 60 Percentile	61 to 80 Percentile	81 to 100 Percentile
Wave Exposure	0 to 20 Percentile	21 to 40 Percentile	41 to 60 Percentile	61 to 80 Percentile	81 to 100 Percentile
Wind Exposure	0 to 20 Percentile	21 to 40 Percentile	41 to 60 Percentile	61 to 80 Percentile	81 to 100 Percentile
Surge	0 to 20 Percentile	21 to 40 Percentile	41 to 60 Percentile	61 to 80 Percentile	81 to 100 Percentile

Geomorphology and natural habitat rankings were adopted using the ranking scheme suggested by the InVEST user guide [22] which is similar to the one proposed by Hammar-Klose and Thieler [23]. Relief rankings were assigned based on the average elevation of the land area within 5 km of each shoreline segment in five equal bins of 20 percent each. The model estimated the relative wave exposure of each shoreline segment by computing the maximum of the weighted average power of oceanic waves and locally wind-generated waves [22]. Relative wave exposure values were re-ranked as a percentile and rankings of 1 to 5 were assigned to each shoreline segment based on binned percentile values of 20 percent each. Wind exposure for shoreline segments was computed by first dividing the 360 degrees compass rose into sixteen equiangular sectors. Then, for each sector, the average wind speed of the highest 10% wind speeds on record in each sector was multiplied by the fetch distance of that sector and by the percent of all wind speeds on record that blow in the direction of that sector [22]. The values for all sectors of a shoreline segment were then summed and re-ranked as a percentile. Rankings of 1 to 5 were assigned to each shoreline segment based on binned percentile values of 20 percent each. Storm surge elevation is a function of wind speed and direction, and the amount of time wind blows over relatively shallow water. In general, the longer the distance between the coastline and the edge of the continental shelf during a storm, the higher the potential storm surge. The model calculated surge potential by computing the distance of all segments from the shoreline to a specified depth contour. We used a depth of 10 meters which represented the edges of deeply dredged shipping channels present in the bay [22].

The model computed the overall exposure index *EI* for each shoreline segment as the geometric mean of all the variable ranks:

$$EI={\left(R_{Geomorphology}R_{Relief}R_{Habitats}R_{WindExposure}R_{WaveExposure}R_{Surge}\right)}^{1/6}$$

The model also computed an erosion index *ErI*, as the geometric mean of selected variables:

$$ErI={\left(R_{Geomorphology}R_{Habitats}R_{WaveExposure}\right)}^{1/3}$$

We assessed shoreline exposure under 3 scenarios: 1) current conditions, using the most recent data available, 2) future conditions, based on the high-estimate 90^th^ percentile sea-level rise projections for the year 2080, and 3) historic conditions based on the year 1609, the date of Henry Hudson’s arrival in America. The default InVEST coastal vulnerability model allows the user to include a 7^th^ variable, sea-level rise. We did not predict shoreline exposure using this variable because it required a dataset that contained heterogeneous sea-level rise within the area of interest. To our knowledge, such a dataset does not yet exist for Jamaica Bay. We instead accounted for sea-level rise by mapping future inundation under the assumptions of homogenous sea-level rise, no marsh accretion or subsidence, and no loss of marshland to forces other than sea-level rise.

We used ArcGIS 10.3 to prepare our datasets for input into the model. Our area of interest for all scenarios was primarily composed of the shoreline segments that fell within the Jamaica Bay-Rockaway Inlet Hydrologic Unit (HUC 0203020201), as delineated by the United States Geological Survey’s (USGS) Watershed Boundary Dataset, expanded to include the ocean side, southern shoreline of the Rockaway Peninsula. All scenarios used the same climatic dataset derived from the National Oceanic and Atmospheric Administration’s (NOAA) WAVEWATCH III model. This dataset was used to determine the wave exposure, wind exposure, and surge potential rankings for each shoreline segment. This dataset reflects current climate patterns so our analysis does not account for climate changes that have occurred since 1609 or will occur by 2080. Results from all scenarios were at the 80 m^2^ resolution as that was the finest resolution at which all three scenarios would run.

For our current conditions scenario, we used the USGS national elevation dataset (NED) 1/3^rd^ arc second resolution (∼10 m^2^) digital elevation model (DEM) as our topography dataset. We created a 60 m^2^ bathymetric DEM by interpolating bathymetric soundings points from NOAA’s Electronic Navigational Charts (ENC) using the empirical Bayesian kriging method. We created a shoreline geomorphology dataset using data from United States Fish and Wildlife Service (USFWS) National Wetlands Inventory dataset (NWI) to classify sandy, cobble/gravel, and emergent wetland shorelines and interpreted high resolution (0.35 m^2^), post Hurricane Sandy, leaf off imagery and Bing maps bird’s-eye view, 45-degree oblique imagery to classify rip-rap, pier, and bulkhead shorelines (Table 2). Habitat cover datasets were derived from a high resolution (∼3 m^2^) ecological cover map developed by the Natural Areas Conservancy (NAC) [24], and supplemented using the NWI dataset (Table 3). The aquatic bed, high dune, and low dune classes were extracted from the NWI dataset. All other classes were extracted from the NAC dataset.

**Table 2:** Shoreline geomorphology rankings as an aggregate of all possible classes across all three temporal scenarios.

Shoreline geomorphology Class	Rank
Bulkhead	2
Pier	3
Previously developed	3
Rip-rap	3
Emergent wetland	4
Cobble/gravel shore	4
Exposed soil	5
Sandy shore	5

**Table 3:** Habitat cover class rankings and protective distances as an aggregate of all possible classes across all three temporal scenarios.

Habitat class	Rank	Protective distance (m)
High saltmarsh	1	400
Low saltmarsh	2	75
Eastern North American wet meadow	2	400
Brackish tidal marsh	2	125
Eastern North American freshwater marsh	2	125
High dunes	2	125
Northern and central ruderal wet meadow and marsh	3	100
Northern and central shrub swamp	3	75
Northern and central ruderal meadow and shrubland	3	100
Northern and central sand barrens group	3	50
Eastern coastal beach group	3	65
Northern Atlantic dune and coastal grassland and shrubland	3	50
Maritime shrubland and successional maritime forest	3	75
Low dunes	3	75
Aquatic beds	4	1200

We prepared datasets for the future scenario assuming 1.47 m of sea-level rise, the high-estimate 90^th^ percentile sea-level rise projections for New York City in the year 2080 [25]. The shoreline for this scenario was delineated using the 1.47 m contour line of the NED DEM. Shoreline geomorphology was reassessed and two additional categories were added for this scenario: previously developed shorelines, where water breached infrastructure, and exposed soil, where the future shoreline fell on a current grass field (Table 2). The habitat cover datasets were clipped to reflect habitat loss due to inundation.

Input datasets for the past (1609) scenario were developed using data from the Welikia Project developed by the Wildlife Conservation Society (WCS). This project aimed to map Jamaica Bay’s topography, bathymetry, and habitat cover, as they likely existed during Henry Hudson’s 1609 voyage to the region, before European settlement in the area. There are notable differences between the modern state of Jamaica Bay and the hypothesized Jamaica Bay of 1609. In an analysis of 100 maps and charts from 1501 to 1844, with careful consideration given to map scale and historical context, Sanderson (2016) presented a hypothesis regarding the east-to-west progression of the Rockaway Peninsula and the subsequent formation of the Bay’s interior marsh islands approximately 200–230 years ago due to a decrease in oceanic wave action [26].

Shoreline geomorphology and habitat cover classes were assigned ranks to reflect their ability to protect shoreline segments from exposure and erosion where highly protective segments were given ranks of 1 and highly exposed segments were given ranks of 5 (Tables 2 and 3). Habitat classes were assigned protective distances based on the geomorphological characteristics of each habitat. For example, very low lying, low sloping habitats, such as low saltmarsh and aquatic beds, were given a high protective distance since wave action would flow over these habitats during a longer period of time and to a larger extent (Table 3).

The model’s final outputs consisted of raster maps where the cell values represented shoreline segments and were encoded with nine metrics at the segment level including: geomorphology, relief, natural habitats, wave exposure, and surge potential rankings as well as an overall coastal exposure index value, the exposure index value if habitats were not present, the habitat role value (the difference between the exposure index value and the no habitat exposure index value), and the erodibility status of the segment (1 = erodible, 0 = not erodible) as determined by the erosion index.

We assigned a primary stakeholder to each shoreline segment to enable analysis of current vulnerability by stakeholder. Stakeholder data were obtained from the New York City Department of City Planning PLUTO dataset, a land use and geographic dataset which identifies ownership of tax lots in New York City. Stakeholder classifications include: JFK Airport, public lands, public lands (NYC only), private lands, Breezy Point Cooperative, Gateway National Recreation Area, and US Army (Fort Hamilton). A shoreline segment’s primary stakeholder was determined using the guiding question “if this segment of shoreline fails (i.e. breached, inundated, eroded) what group will be primarily impacted?” As a result of this protocol, a shoreline segment’s primary stakeholder may differ from the actual owner of the parcel directly adjacent to that segment. For example, in select areas of the bay, the Belt parkway abuts the shoreline forming a narrow boundary between the water and other properties. Although such segments would be owned and managed by the Department of Transportation, if they were breached, the properties beyond the parkway (such as a group of private residences) would be most impacted and would therefore be considered the primary stakeholder.

In addition, we compared the coastal vulnerability results from our current scenarios for exposure and surge against mapped areas of Jamaica Bay affected by Hurricane Sandy. Our data was created by comparing pre- and post-Sandy aerial photos in ArcGIS 10.3. Pre-Sandy imagery was obtained on March 1, 2012 by USGS; Jamaica Bay was flown post-Sandy on November 3 and 4, 2012 by the Federal Emergency Management Agency (FEMA). We assigned metrics based on visible degradation present in the post-Sandy photos using the 60–40 rule [27]. The metrics assessed were: minimal, moderate and severe flooding, minimal, moderate and severe artificial debris, minimal and moderate natural debris (no severe natural debris was present), sand deposition, and marsh dieback (distinct brown patches) [28]. Flooding was defined as areas with clear changes in water content not driven by tides. This was apparent as dark sunken areas in grass or marsh and on pavement and developed lands. Flood size delineation was determined by sheer area of the flood including channel and pond expansion, creation or scouring [29]. If a flood covered only one cell (100 m x 100 m) it was digitized as minimal flooding. A flood with a size up to nine contiguous cells was classified as moderate flooding and anything larger was classified as severe flooding. Artificial debris (e.g. human infrastructure) and natural debris (e.g. accumulation of wrack) are types of depositional events [30]. Artificial debris was defined as anything that was not sand, rip-rap, or natural. Most of this material consisted of small piles of trash and rubble, boats, cars, and structural materials. Trash, rubble, car parts, and siding were defined as minimal artificial debris. Cars and boats were defined as moderate artificial debris. Large artificial debris was classified as anything larger than a boat or car such as a dock, mooring or house. Natural debris was limited to trees, brush and wrack. Areas of the beach where natural debris had accumulated (i.e. brush and wrack) were labeled as minimal natural debris because the individual masses were never very large, however the collective mass of these groupings could cover considerable areas. In this case the entire area was digitized rather than individual groupings. When present, downed trees were defined as moderate natural debris. We found no situations where large natural debris accumulated and thus have no severe natural debris category. As the storm’s surge came through, much of the beach sand located on the southern edge of the study area was pushed into neighboring areas. If this occurred, the area was digitized and classified as sand deposition. Marsh dieback was defined as areas that showed clear evidence of plant death due to the storm (browned areas of dead vegetation) [29]. This was a difficult attribute to see in the imagery and was used sparingly. Damage maps were reviewed by the New York City Department of Parks and Recreation for consistency and errors. Areas of change in known restoration sites within the time period between our two sets of aerial photos were assumed to be the result of restoration, not damage and were removed from the damage dataset. Likewise, areas of change in known sand placement sites by the US Army Corps of Engineers (USACE) were assumed to be the result of USACE activities, not sand deposition and were again removed.

Data were assembled and loaded in SAS 9.3 for statistical analysis. Several questions were asked of the data: 1) are there significant differences in the nine predicted coastal vulnerability metrics among time periods (past, current and future)? General linear models (PROC GLM) were used to ascertain the differences by vulnerability metric with Tukey-Kramer utilized to determine which time periods were significantly different from each other; 2) are there significant differences in current coastal vulnerability for each of the nine metrics depending on stakeholder type? General linear models were again used to ascertain the statistical significance of the relationship between vulnerability and stakeholder type; 3) are there significant correlations between current coastal vulnerability metrics (overall coastal exposure index and surge) and Hurricane Sandy damage effects such that damage can be used as a surrogate for vulnerability? Pearson correlations were used to ascertain the strength of the relationship between selected vulnerability metrics and distance to damage. Means and standard deviations are noted in the text and tables. Except where noted, significant differences were determined at p < 0.05.

Spatially, we used the Moran’s I spatial autocorrelation tool in ArcGIS 10.3 to test for significant clustering in the coastal vulnerability results for each time period. We then did a hot spot analysis of coastal exposure. The optimized hot spot analysis geoprocessing tool identifies the appropriate scale of analysis and corrects for spatial dependence. The optimized hot spot analysis returns Gi* z-scores, p-values and binned confidence levels for each feature with respect to its analysis value. In the context of this study, higher positive Gi* z-scores and low p-values indicate vulnerable coastal locations. Conversely, high negative Gi* z-scores and low p-values indicate locations with low coastal vulnerability.

## Results

**Fig 2:**
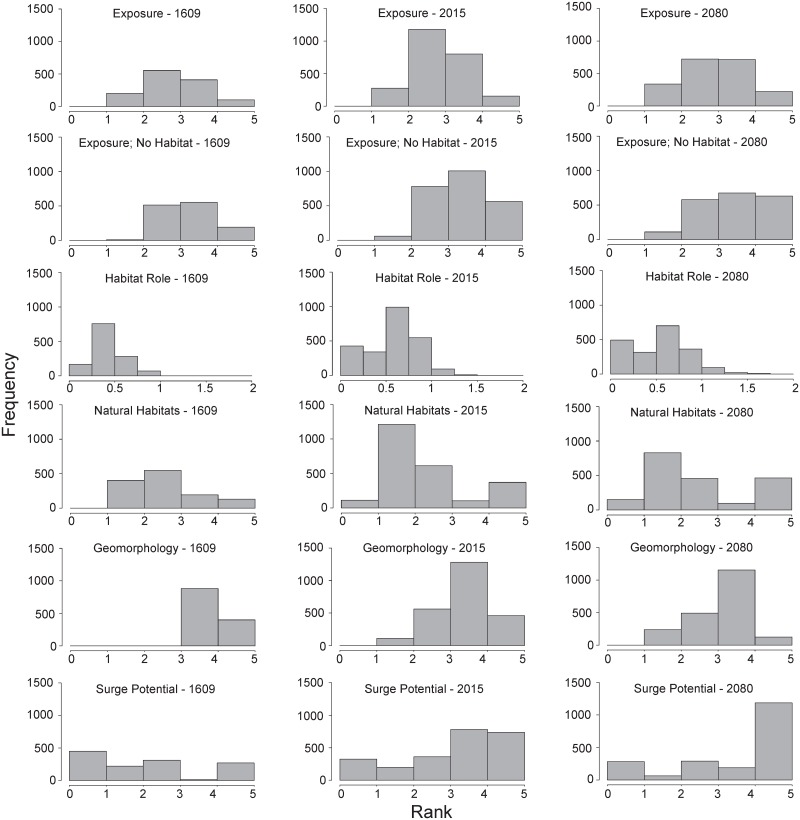
Histograms showing ranks of selected metrics. Ranks vary from very low exposure (rank = 1) to very high exposure (rank = 5). The ‘habitat role’ value is the difference between ‘exposure’ and ‘exposure; no habitat’ at each shoreline segment. Relief and wave exposure were not included since there was an equal frequency of segments in each rank for those metrics. Erosion were not included and reported elsewhere as percentage values.

Frequency distributions of the nine vulnerability metrics from the current scenario showed a normal pattern with the bulk of the values ranging from 2–4 for all metrics except erodibility which only produces values of 0 or 1, habitat role which ranged from 0–1, geomorphology, wave exposure and surge whose values ranged from 4–5, and relief which ranged almost equally across all values (lower values for all metrics indicate decreased vulnerability) (Fig 2). Similar patterns emerged for the past and future scenarios with a few notable exceptions. Past natural habitat values mostly ranged from 1.5–2.5 and past surge and wave exposure ranged across all values. Future surge was predicted to be at or near 5 for the majority of values and a lower percentage of erodibility values were predicted to be 1 (6.2% compared with 19.1% and 31.2% for current and past scenarios, respectively).

We found significant differences in vulnerability between past, current and future scenarios for all nine metrics except relief and wave exposure (Table 4). Of the significant metrics, geomorphology, erodible shoreline, and natural habitats were the only metrics with highest means for the past time period. The remaining significant metrics had lowest means for the past time period. Of the significant metrics, geomorphology, habitat role, and erodible shoreline had higher means for the current time period compared to the future time period. All other significant metrics had higher means for the future time period compared to the current time period. All vulnerability metrics had significantly differing means across all time periods except natural habitats for which past and future did not differ, exposure no habitat for which current and future did not differ, and exposure for which past and current did not differ.

**Table 4:** Coastal vulnerability comparison using GLM between past, current and future scenarios.

Vulnerability model	F-value	p-value	Past-Current	Current-Future	Past-Future	Past mean	Current mean	Future mean
Relief	0.00	0.9995				3.00	3.00	3.00
Wave exposure	0.00	0.9995				3.00	3.00	3.00
Geomorphology	412.51^a^	<0.0001 ^a^	*	*	*	4.31	3.87	3.58
Natural habitats	33.85 ^a^	<0.0001 ^a^	*	*		2.56	2.25	2.52
Exposure no habitat	37.73 ^a^	<0.0001 ^a^	*		*	3.23	3.41	3.46
Exposure	13.45 ^a^	<0.0001 ^a^		*	*	2.81	2.85	2.94
Habitat role	106.62 ^a^	<0.0001 ^a^	*	*	*	0.41	0.56	0.52
Erodible shoreline	189.03 ^a^	<0.0001 ^a^	*	*	*	0.31	0.19	0.06
Surge	395.87 ^a^	<0.0001 ^a^	*	*	*	2.55	3.59	3.96

Mean values run from 0–5 and are unitless.

^a^ Results are significant.

* Significant differences in means.

**Fig 3:**
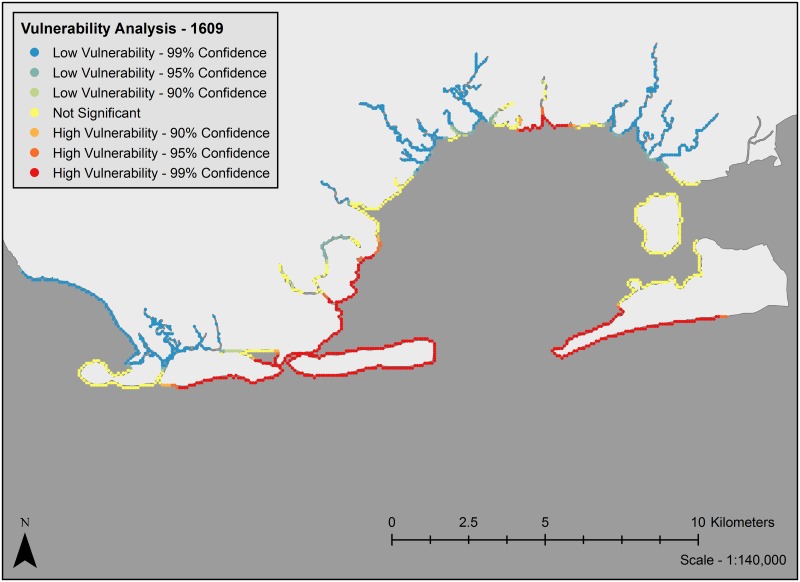
Vulnerability map of coastal exposure vulnerability for past conditions. Landmass polygon obtained from the Wildlife Conservation Society, Welikia Project.

**Fig 4:**
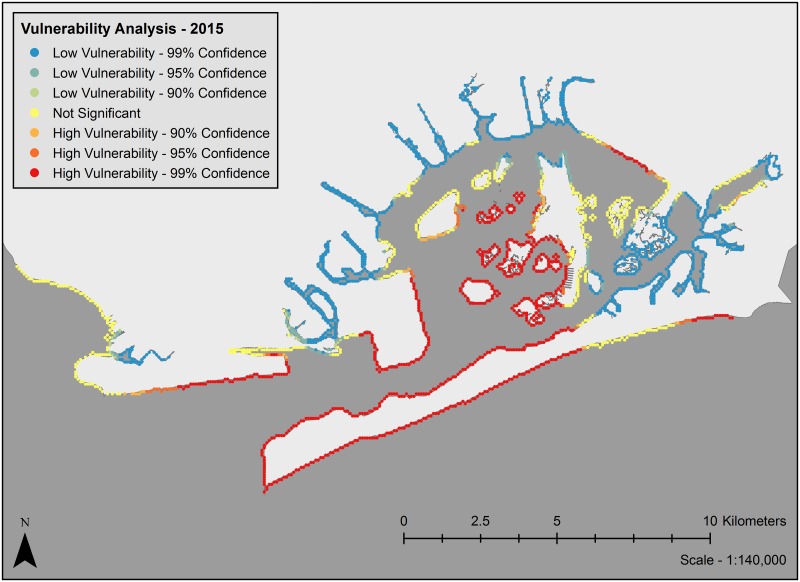
Vulnerability map of coastal exposure vulnerability for current conditions. Landmass polygon delineated using the USGS NED 1/3rd arc second n41w074 and n41w075 digital elevation model.

**Fig 5:**
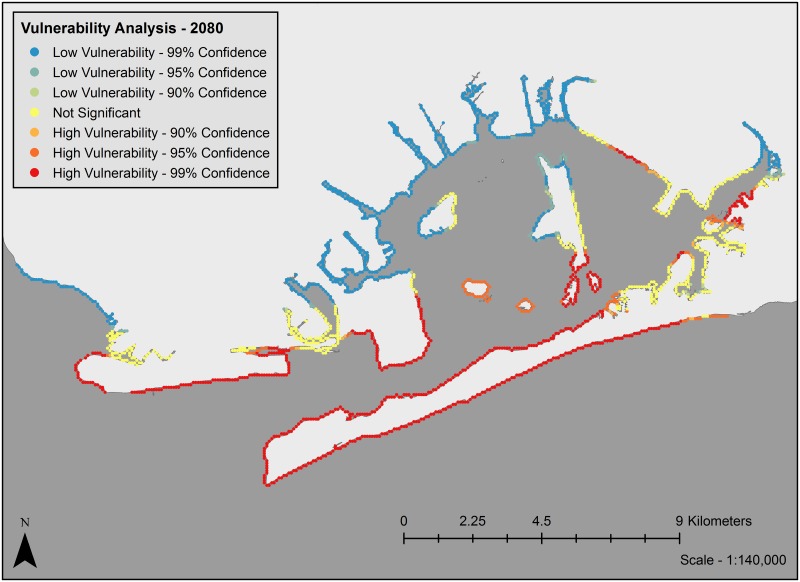
Vulnerability map of coastal exposure vulnerability for future conditions. Landmass polygon delineated using the USGS NED 1/3rd arc second n41w074 and n41w075 digital elevation model.

Analysis of clustering of vulnerability metrics using Moran’s I showed significant clustering of coastal exposure values for past, current and future scenarios (z-score = 54.59, p < 0.0001, z-score = 80.35, p < 0.0001, and z-score = 90.62, p < 0.0001, respectively). Vulnerability maps (Figs 3–5) show that under current conditions, many of the marsh islands in Jamaica Bay are highly vulnerable along with a large portion of the shoreline of the Rockaway Peninsula and areas along the western edge near the entrance to the bay. Most of the protected shoreline areas along the northern, southeastern and northwestern edges of the bay are only minimally vulnerable. The vulnerability map for future conditions is fairly different. Many marsh islands are predicted to disappear, so few marsh islands are still vulnerable. With the protection of the marsh islands gone, areas of the southeastern edges of the bay are now moderately vulnerable, in particular the southern edges of JFK Airport and the northern bay side of the Far Rockaways. The entirety of the Rockaway Peninsula is now either moderately or highly vulnerable however sections of the northern parts of the bay are less vulnerable. The vulnerability map for past conditions is also quite different. No marsh islands existed and the entrance to the bay was from the south where today the Rockaway Peninsula sits. The most vulnerable sections are near the entrance to the bay and the northern shoreline of the bay most likely impacted by wave action through the mouth of the bay. The northeast and northwest sections within the bay are protected and thus least likely to be vulnerable.

We tested the likelihood for vulnerability to differ across stakeholder categories by comparing the means of vulnerability metrics by stakeholder for the current scenario using GLM (Table 5). We found significant differences in vulnerability for all nine vulnerability metrics. In general, Breezy Point Cooperative had the highest mean vulnerability rankings across all vulnerability metrics except natural habitats (US Army highest) and habitat role (Gateway National Recreation Area highest). In general, US Army lands had the lowest mean vulnerability rankings across all vulnerability metrics except wave exposure, natural habitats and exposure (public lands NYC lowest) and exposure no habitat and surge (JFK Sirport lowest). Mean public lands in NYC and US Army lands both had lowest vulnerability for erodible shorelines.

**Table 5:** Results of comparison using GLM of vulnerability metrics under the current scenario across stakeholder types.

Vulnerability model	F-value	p-value	Mean Breezy Point Cooperative	Mean Gateway National Recreation Area	Mean JFK Airport	Mean private lands	Mean public lands	Mean public lands (NYC only)	Mean US army
Relief	31.21^a^	<0.0001 ^a^	3.59^b^	3.34	2.29	2.68	2.79	3.33	1.00 ^c^
Wave exposure	41.71 ^a^	<0.0001 ^a^	5.00 ^b^	3.27	2.78	2.39	2.91	1.83^c^	4.09
Geomorphology	193.22 ^a^	<0.0001 ^a^	5.00 ^b^	4.17	3.73	3.10	3.90	3.17	3.00 ^c^
Natural habitats	87.43 ^a^	<0.0001 ^a^	3.10	1.92	2.28	3.18	1.99	1.56 ^c^	5.00^b^
Exposure no habitat	132.16 ^a^	<0.0001 ^a^	4.46 ^b^	3.73	2.94 ^c^	2.97	3.24	3.06	3.00
Exposure	67.87 ^a^	<0.0001 ^a^	4.01 ^b^	3.05	2.44	2.65	2.66	2.42 ^c^	3.00
Habitat role	120.66 ^a^	<0.0001 ^a^	0.45	0.68 ^b^	0.50	0.32	0.57	0.64	0.00 ^c^
Erodible shoreline	60.22 ^a^	<0.0001 ^a^	1.00 ^b^	0.25	0.07	0.01	0.22	0.00 ^c^	0.00^c^
Surge	43.56 ^a^	<0.0001 ^a^	4.00 ^b^	3.92	2.29 ^c^	3.40	3.42	3.00	4.00^b^

^a^ Results are significant.

^b^ Maximum mean value among stakeholders.

^c^ Minimum mean value among stakeholders.

We also looked at whether damage from Hurricane Sandy could be used as a surrogate for vulnerability and the strength of the relationship between distance to noted damage and vulnerability (Table 6). Correlations between the vulnerability values of two metrics (surge and exposure) and their distances in meters to five major damage groupings revealed that significant correlations exist for all vulnerability-damage combinations except exposure and distance to artificial debris. All vulnerability metrics were inversely correlated with damage except for surge and distance to natural debris, and both exposure and surge and distance to artificial debris. The strongest correlation was an inverse relationship between exposure and distance to marsh dieback (r = -0.25, p <0.0001).

**Table 6:** Correlations between damage type and selected coastal vulnerability metrics for the current scenario.

	Distance to sand deposition		Distance to natural debris		Distance to flooding		Distance to marsh dieback		Distance to artificial debris	
Vulnerability model	r	p-value	r	p-value	r	p-value	r	p-value	r	p-value
Exposure	-0.08327^a^	<0.0001 ^a^	-0.12021 ^a^	<0.0001 ^a^	-0.22322 ^a^	<0.0001 ^a^	-0.25484 ^a^	<0.0001 ^a^	0.00637	0.7541
Surge	-0.04794 ^a^	0.0184 ^a^	0.17855 ^a^	<0.0001 ^a^	-0.06241 ^a^	0.0021 ^a^	-0.23481 ^a^	<0.0001 ^a^	0.15355 ^a^	<0.0001 ^a^

^a^ Results are significant.

## Discussion

In this study, spatial and temporal changes in nine metrics of coastal vulnerability were investigated along the shoreline of Jamaica Bay, New York for past, current and future time periods. This characterization is especially timely given ongoing threats from climate change including sea-level rise and shoreline erosion [9,25]. We found significant differences in vulnerability metrics between past, current and future scenarios for all nine metrics except relief and wave exposure. Overall exposure to coastal erosion in Jamaica Bay is expected to increase through time. The mean values for relief and wave exposure remained the same for all three temporal scenarios (rank of 3) due to the metric’s ranking scheme (Table 1). These two variables are ranked by percentile values so an equal number of segments were assigned to each rank from one to five. Although natural habitats covered Jamaica Bay almost entirely in 1609, with the exception of a few small Native American settlements, mean natural habitat values were predicted to be higher (less protective) in the past scenario than in the current and future scenarios. Differences in input data quality between the scenarios and differences in the spatial structure and distribution of habitats between the past and future may have contributed to this result. First, the thematic resolution of our habitat cover data was much lower in the past scenario compared to our current and future scenarios; five unique habitat types were present in the past compared to 15 types for our current and future scenarios. The model ensures that segments with only one type of unique habitat are ranked higher than those with multiple habitat types. Second, the extent of coastal habitat zones differs greatly between the past scenario and the other scenarios. Under current and future conditions, one could profile the entire suite of coastal zones from low marsh to upland habitat in a much shorter distance from the shoreline when compared to past conditions in which coastal habitat zones stretched over very wide extents. A much longer profile would be needed to capture all coastal habitat zones for the past scenario. Due to these factors, shoreline segments in the current and future scenarios were often determined to be protected by more habitat types than segments in the past scenario. These factors may also demonstrate the limitations of this model in comparing a very urbanized coastal system to an undeveloped one. Alternatively, natural habitat cover may indeed be more protective now compared to the past due to the formation of the interior marsh islands. These islands were often close enough to the peripheral shoreline segments for the model to classify them as effective protective habitat.

The mean estimated surge ranking increased through time. We attribute the low surge value in the past scenario to the lack of dredged channels in the bay in precolonial times. We attribute the increase in the mean surge value between the past and future scenarios to the greater fetch distance expected with the loss of interior marsh islands to sea-level rise. The Intergovernmental Panel on Climate Change (IPCC) has also reported that it is likely that there has been an increase in extreme sea-levels, as experienced during storm surges, since 1970, being mainly the result of rising mean sea-level [31]. It should also be noted that we used the same wind and wave datasets for all three scenarios so the model did not account for any differences in wind and wave patterns that may have presented in 1609 or that may occur by 2080; the findings with respect to surge only represent changes in geomorphology.

Our model also predicted that the percent of shoreline segments determined to be erodible fell through time from 31.2% to 19.1% to 6.2%. This is partially due to the anthropogenic hardening of the shoreline over time. In 1609 the shoreline of the entire bay was naturalized with sandy shorelines and marsh ecosystems. According to our aerial imagery interpretation analysis of shoreline geomorphology we estimated that by 2015 29% of the shoreline was hardened (rip-rap, bulkhead, or pier). Assuming no further anthropogenic alteration of the shoreline, no loss of marshland to forces other than sea-level rise, and no marsh accretion, the percent of hardened shoreline is expected to increase to 40% due to sea-level rise alone by 2080. This is because natural shoreline segments, such as the low lying marsh islands, are likely to be inundated by sea-level rise while hardened shorelines are often tall enough that they will not likely be inundated by 2080. While these hardened shorelines are less erodible than natural shorelines, they do not provide myriad of other environmental services that natural shorelines can provide [1–3]. The science of sea-level projections is rapidly evolving and our assumed 1.47 m sea-level rise is higher than rates proposed since the time of this analysis [32]. It is important that future research reflect the most recent sea-level rise rates as the science continues to evolve. While we assumed no accretion of saltmarsh soils in this study, the future response of saltmarshes to sea-level rise is a contested subject. Some studies predict that future accretion will accelerate at sufficient rate to keep pace with sea-level rise [33]. While more research into the accretion rates of Jamaica Bay are required, the current accretion rate of the saltmarshes of the Tuckerton Peninsula, New Jersey, approximately 80 miles south of Jamaica Bay, is 0.17 cm per year [34]. This rate is well below current sea-level rise rates, and previous studies document a clear and significant loss of saltmarsh coverage in Jamaica Bay during the last few decades [9,12,13,14]. Jamaica Bay’s saltmarshes may erode laterally due to erosional forces regardless of vertical forces of sea-level rise and accretion. This model also does not account for disruption in sand transport and deposition patterns due to urbanization and the introduction of coastal infrastructure since 1609 [35], or the effects hardened shorelines may have on adjacent shorelines, such as flanking erosion to adjacent natural shorelines due to reflectance of wave energy [17].

The geography of vulnerable areas in the bay shifts with time. In 1609, areas of high vulnerability (on the barrier islands and in the area across from the bay’s mouth) largely reflect direct exposure to oceanic waves (Fig 3). Although it is difficult to compare the past scenario with the current and future scenarios due to the drastic changes in the geography of the bay as a whole, some clear trends emerge when comparing the current and future scenarios of the bay. Currently, the interior marsh islands, Floyd Bennett Field, the Rockaway Peninsula, and the portion of Coney Island at the mouth of the bay are low vulnerability areas while the northern and eastern periphery of the bay are largely exposure cold spots (Fig 4). By 2080, many of the vulnerable marsh islands will have shrunk or been inundated entirely and the northern and eastern areas of the bay will become more vulnerable (Fig 5). While currently the uninhabited marsh islands bear the brunt of erosional forces, by 2080 high vulnerability locations will likely shift to shorelines that front residential and other built areas such as the Far Rockaways, the eastern portion of the Rockaway Peninsula. This will likely have implications economically, structurally and socially [36].

Our stakeholder analysis found clear differences in vulnerability between the various stakeholders in the bay (Table 5). The Breezy Point cooperative had highest vulnerability rankings for most categories and the highest overall exposure ranking. One factor that contributed to this result is the Cooperative’s location on the Rockaway inlet, which is exposed to open ocean on one side, is entirely sandy shoreline, has low average relief, and is close to a large dredged channel on its bay side. 100% of the cooperative’s shoreline was ranked as erodible in 2015, far more than any other stakeholder. The US Army had the highest vulnerability ranking for natural habitat and the lowest for geomorphology since all shorelines assigned to them are completely bulkheaded. Natural habitats play the biggest role in protecting shorelines assigned to the Gateway National Recreation Area and other public lands. This result is unsurprising as many of these areas have been managed as natural preserves. John F. Kennedy International Airport had the lowest vulnerability for surge of any stakeholder. This can be attributed to the abundance of marsh islands between the mouth of the bay and the airport and the lack of dredged channels on the eastern edge of the bay.

Our damage correlation analysis compared distance to damage due to Hurricane Sandy to the model’s predicted overall exposure and surge values (Table 6). Significant correlations exist for all vulnerability (exposure/surge)-damage combinations except for exposure and distance to artificial debris. Most correlations were weak but the strongest was the inverse relationship between exposure and distance to marsh dieback. As such, our results do not support the use of distance to damage as a surrogate for vulnerability. These correlations may have been weak due to limitations in our analysis. We determined distance from each shoreline segment to the nearest area of storm damage. This resulted in some segments being paired with damage areas that most likely weren’t related, such as ones across open water or at great distances. It is difficult to attribute any given area of damage to any single section of shoreline. A more robust analysis would need to determine a shoreline segments ‘damage-shed’, the exact areas which would face damage through the failure of specific shoreline segments.

Communities in coastal areas around the world will likely feel the effects of climate change through subsequent sea-level rise and increased storm events [31,35] which may strongly affect cultural and natural resources. Coastal protective features, ranging from storm surge barriers and levees to natural features (e.g. wetlands), have been promoted in coastal areas by the USACE, environmental groups, and state and local government to decrease future flood risk. However, it is not understood where these measures might be best implemented to affect the most benefit. Coastal areas of the United States are often unique couplings of natural wetland habitat with densely populated urban landscapes. The juxtaposition of heterogeneous shoreline habitat creates variability in coastal vulnerability rankings. Resource managers are thus increasingly using ecosystem services modeling in the creation of management plans and restoration efforts. As demonstrated here, coastal vulnerability results can be coupled with additional data (e.g. stakeholders) and modeled over multiple time periods to identify the extent and severity of change and impacts on other entities. Data from past time periods can be used to better understand how changes in the landscape may have influenced current coastal vulnerability and data on future scenarios can be used to predict future changes in vulnerability. Further, these methods can be tailored for any coastal region for which data exist. Thus, our methods have considerable potential to provide valuable predictions of coastal vulnerability to resource managers to effectively identify areas for restoration and protection.

## Supporting Information

S1 File Raw results and supplementary data as shapefiles. The results of the InVEST vulnerability models and the Moran’s I spatial autocorrelation analysis as ESRI shapefiles, as well as map identifying local Hurricane Sandy damage.
(ZIP)

S1 Table Initial Datasets. The datasets used for this analysis, the source organization, notes on how they were used, and a web location of the data when available. All but one dataset (New York City Ecological Cover Map) are publically available.
(XLSX)

S2 Table Raw results. The results of the InVEST vulnerability models. Each record represents a shoreline segment and include geographic coordinates.
(XLSX)
